# Single-Cell Transcriptional Response of the Placenta to the Ablation of Caveolin-1: Insights into the Adaptive Regulation of Brain–Placental Axis in Mice

**DOI:** 10.3390/cells13030215

**Published:** 2024-01-24

**Authors:** Maliha Islam, Susanta K. Behura

**Affiliations:** 1 Division of Animal Sciences, University of Missouri, Columbia, MO 65211, USA; mifcr@missouri.edu; 2 MU Institute for Data Science and Informatics, University of Missouri, Columbia, MO 65211, USA; 3 Interdisciplinary Reproduction and Health Group, University of Missouri, Columbia, MO 65211, USA; 4 Interdisciplinary Neuroscience Program, University of Missouri, Columbia, MO 65211, USA

**Keywords:** trophoblast, neuron, glia, proliferation, Caveolin, placenta, embryo

## Abstract

Caveolin-1 (*Cav1*) is a major plasma membrane protein that plays important functions in cellular metabolism, proliferation, and senescence. Mice lacking *Cav1* show abnormal gene expression in the fetal brain. Though evidence for placental influence on brain development is emerging, whether the ablation of *Cav1* affects the regulation of the brain–placental axis remains unexamined. The current study tests the hypothesis that gene expression changes in specific cells of the placenta and the fetal brain are linked to the deregulation of the brain–placental axis in *Cav1*-null mice. By performing single-nuclei RNA sequencing (snRNA-seq) analyses, we show that the abundance of the extravillious trophoblast (EVT) and stromal cells, but not the cytotrophoblast (CTB) or syncytiotrophoblast (STB), are significantly impacted due to *Cav1* ablation in mice. Interestingly, specific genes related to brain development and neurogenesis were significantly differentially expressed in trophoblast cells due to *Cav1* deletion. Comparison of single-cell gene expression between the placenta and the fetal brain further showed that specific genes such as plexin A1 (*Plxna1*), phosphatase and actin regulator 1 (*Phactr1*) and amyloid precursor-like protein 2 (*Aplp2*) were differentially expressed between the EVT and STB cells of the placenta, and also, between the radial glia and ependymal cells of the fetal brain. Bulk RNA-seq analysis of the whole placenta and the fetal brain further identified genes differentially expressed in a similar manner between the placenta and the fetal brain due to the absence of *Cav1*. The deconvolution of reference cell types from the bulk RNA-seq data further showed that the loss of *Cav1* impacted the abundance of EVT cells relative to the stromal cells in the placenta, and that of the glia cells relative to the neuronal cells in the fetal brain. Together, the results of this study suggest that the ablation of *Cav1* causes deregulated gene expression in specific cell types of the placenta and the fetal brain in mice.

## 1. Introduction

In mammalian reproduction, the placenta plays key roles in the development of the fetus [1]. The deregulation of placental functions impacts the developmental programs of the offspring [2]. The brain development of the fetus is intricately reliant on placental functions during normal as well as adverse pregnancy conditions [3,4,5]. Earlier, we showed that the placenta and fetal brain express genes in specific patterns in mice [6]. The same study further showed that the placenta expresses different ligand genes when the uterus lacks Forkhead box 2 (*Foxa2*), a gene required for pregnancy establishment in mice, supporting the idea that placental functions are adaptive [6]. Placental adaptive functions protect the fetal brain from adverse maternal conditions including obesity, infection, malnutrition, smoking, and substance use [7].

In mice, neural tubes are formed around embryonic day 9 (d9) [8]. Choroid plexus formation begins on day 12 when the medial, lateral and caudal ganglionic sections as well as the hypothalamus grow rapidly [9]. On d15, the fetal brain shows the highest level of microglia invasion with all the six layers of the cerebral cortex being distinctive in development [10]. When the formation of the neural tube begins in the embryo, the placental labyrinthine zone begins to develop in a rapid manner [10,11]. On day 15, the placental labyrinth zone becomes fully functional with the syncytiotrophoblast, and the choroid plexus and hypothalamus are developed in the fetal brain [10]. In the placenta, the syncytiotrophoblast cells and the extravillous trophoblast cells are differentiated from cytotrophoblast cells. In the developing central nervous system, the differentiation of brain cells is accomplished by complex temporal and spatial cellular differentiation processes. These parallel developmental milestones are also associated with coordinated gene expression changes in the placenta and fetal brain on day 15 [6].

Developmental defects in the brain due to a dysfunctional placenta can lead to different neuropsychiatric disorders later in life in humans [12], supporting the idea that the brain–placental axis plays a vital role in the fetal programming of development [4]. The brain–placental axis refers to the molecular and developmental links of the fetal brain with the placenta [4,13,14]. The deregulation of these links between the placenta and the fetus can cause developmental defects in the offspring brain [3,5,12,15,16]. In mice, the placenta and fetal brain express different genes in specific patterns [6]. The conditional knockout of transcriptional repressor *Rest* (RE1 silencing transcription factor) in the mouse placenta leads to deregulated gene expression in the offspring brain [16]. Thus, the gene expression patterns of the placenta and the fetal brain are important in the study of the regulation of the brain–placental axis [4].

Recently, we showed that the ablation of Caveolin-1 (*Cav1*), a key regulator of cell metabolism, proliferation and senescence, was associated with metabolic programming of the brain in mice [17]. By performing single-nuclei RNA sequencing (snRNA-seq), the same study [17] further showed that specific cell types of the fetal brain were deregulated due to the ablation of *Cav1*. A study performed by other researchers has shown that *Cav1*-knockout (KO) in mice leads to increased amyloid beta, hyperphosphorylation of tau, astrogliosis and the decreased cerebrovascular volume of the brain which are hallmarks of Alzheimer’s disease [18]. *Cav1*-null mice also exhibit a drastic reduction in lifespan [19] with abnormal endothelial functions [20], angiogenesis [21], and cellular proliferative and vasculature [20].

In the present study, we hypothesize that specific cell types are associated with the adaptive regulation of the brain–placental axis in mice lacking *Cav1*. To test this hypothesis, the primary aim of this study is to identify the cell types of the placenta that respond to the absence of *Cav1* at the transcriptional level. Another aim of this study is to perform a comparative analysis of gene expression changes of the placenta and the fetal brain to investigate if specific cell types are associated with the adaptive regulation of the brain–placental axis in response to *Cav1* ablation.

## 2. Materials and Methods

### 2.1. Animal Breeding

The wild type (WT: strain C57BL/6J) and *Cav1* knockout mice (KO: strain B6.Cg-*Cav1^tm1Mls^*/J) were obtained from the Jackson Laboratory. Approximately 8-week old mice of both strains were used to establish timed pregnancy separately as described earlier [6]. The vaginal plug was observed to keep a record of the start of pregnancy (day 1). On day 15, the pregnant mice were euthanized, and the fetuses and placentae were collected [22]. They were washed in sterile PBS and snap frozen in liquid nitrogen. Day 15 was chosen for sample collection because the placenta becomes fully formed and functional at this time point [23]. The fetal brain also shows the development of all layers of the cerebral cortex at this time [10]. The development of the cerebral cortex is reliant on placenta function, particularly oxygen transportation to the fetus [24]. Moreover, the placenta and fetal brain express different receptor and ligand genes suggesting a robust regulation of the brain–placental axis at this developmental time [6]. The animal procedures were performed by approved procedures and protocols (Approval #25580) of the Institutional Animal Care and Use Committee of the University of Missouri following the Guide for the Care and Use of Laboratory Animals (National Institutes of Health, Bethesda, MD, USA).

### 2.2. Single-Nuclei RNA-Seq

Single-nuclei RNA sequencing of day-15 placentae (*n* = 2) from the WT and KO mice was performed. The Pure Prep Nuclei Isolation kit (Product No. NUC-201, Sigma, St. Louis, MO, USA) was used to prepare single nuclei as per manufacturer’s instructions. The sample, minced into small pieces on a chilled Petri dish, was added with 2 mL lysis buffer freshly supplemented with 1 M dithiothreitol (DTT) and 10% Triton X-100, and homogenized using a dounce homogenizer. The homogenized mixture was passed through a 70 µm cell strainer. The filtrate after diluting with 3 mL of additional lysis buffer was layered over a freshly prepared 1.8 M sucrose cushion and centrifuged to collect the nuclei pellet. The nuclei pellet was resuspended in 200 µL of ice-cold storage buffer and passed through a 40 µm cell strainer to isolate the single nuclei. A Countess II FL Automated Cell Counter (ThermoFisher, Waltham, MA, USA) was used to count the nuclei. The sequencing libraries were prepared from the nuclei using the 10X Genomics Chromium Single Cell 3′ GEM, Library & Gel Bead Kit v3.1 at the University of Missouri Genomics Technology Core. A Qubit HS DNA kit was used to quantify the libraries. The fragment size was analyzed using an Agilent Fragment Analyzer system. Libraries were sequenced on an Illumina NovaSeq 6000. The sequencing configurations were based on 28 base pairs (bps) on first read and 98 bps on the second read. The libraries were sequenced to a depth of 20,000 paired-end (single-indexing) reads per nucleus. The base call (BCL) files were processed by the *Cell Ranger* pipeline (v. 3.0.1) to generate the FASTQ files. The *STAR* aligner [25] was used to map the reads to the mouse reference genome *GRCm39*. The raw and processed data were submitted to the Gene Expression Omnibus (GEO) database under accession number GSE248126. The snRNA-seq data was analyzed by R package *Seurat* [26]. Cells expressing less than 200 transcripts and more than 20 mitochondrial DNA sequences were excluded from analysis. The normalized filtered data was used for identification of the variable features using the ‘*FindVariableFeatures*’ function in *Seurat*. The scaled-normalized variable feature data was subjected to cluster identification by principal component analysis (PCA) using the ‘*RunPCA’* function with the number of principal components equal to 30. The dimension reduction by Uniform Manifold Approximation and Projection (UMAP) was performed with the first 20 dimensions using the ‘*RunUMAP’* function. The single-cell gene expression neighborhoods were then predicted using the function ‘*FindNeighbors*’ function. Then, cell clusters were identified using the ‘*Find Clusters*’ function where the cluster resolution value was 0.5. Finally, the non-linear dimensional reduction by tSNE (t-distributed stochastic neighbor embedding) [27] was performed using the ‘*RunTSNE*’ function. Marker genes of placental cells were predicted using the ‘*FindAllMarkers*’ function of *Seurat*. The marker genes of placental cells, obtained from the single-cell sequencing resource database *PanglaoDB* [28], were used to annotate the cell types of the predicted clusters.

### 2.3. Bulk RNA-Seq

Placentae of *Cav1* KO mice (*n* = 3) were subjected to bulk RNA-seq analysis as described in our earlier works [17,22,29]. An AllPrep DNA/RNA Mini Kit (Qiagen, Germantown, MD, USA, Cat No./ID: 80204) was used to isolate total RNA. The sample was homogenized with 500 μL RLT buffer (Qiagen, Germantown, MD, USA, Cat No./ID: 79216). The buffer was freshly supplemented with 5 μL of 2-mercaptoethanol. The homogenate was centrifuged for 1 min at ≥8000× *g*. The supernatant was collected to fresh tube and mixed with 1 volume 70% ethanol. RNA was eluted in 30 μL nuclease-free water to a total volume of 60 μL. Concentration of RNA was determined using Nanodrop 1000 spectrophotometer (Thermo Fisher Scientific, Waltham, MA, USA). RNA Integrity Number (RIN) was determined (using Agilent2100) to confirm integrity of RNA (RIN values greater than 7). The sequencing libraries were prepared from the RNA by Novogene Cooperation Inc (Sacramento, CA, USA). Each library was sequenced to 20 million paired end reads of 150 bases using a NovaSeq sequencer. The raw sequence data was processed for quality control (Phred score > 30) using the *fastp* tool [30]. Read alignment to the reference mouse genome (GRCm39) was performed using the *STAR* aligner [25]. The raw and processed data were submitted to GEO database under accession number GSE248093. The gene expression data of the *Cav1*-KO placentae data was compared with that of the WT placentae (accession number GSE157555) generated from our earlier work [22]. Differential gene expression analysis was performed using the R package *edgeR* [31].

### 2.4. Comparison of Gene Expression between Placenta and Fetal Brain Cells

The snRNA-seq data of the placentae from the WT and KO mice generated in the current study were compared with the snRNA-seq data of the fetal brain (accession number GSE214759) generated from our earlier study [17]. Of note, these snRNA-seq data were generated from placentae and fetal brains collected at the same gestation time (day 15) from the same mice strains. In addition, the snRNA-seq of the placenta in the present study and the fetal brain in the previous study was performed in same manner. We sequenced one placenta each from the control and knockout mice in the present study. This approach was also used in the snRNA-seq of the fetal brain in our previous study [17]. We wanted our approach to be consistent in both the studies so that we could compare the placenta and brain data without invoking any bias in the experimental approach. The sequencing of a single sample is also used to compare cells between different animals elsewhere [32]. In the present study, marker genes of the cell types, identified by *Seurat*, were compared between the placentae and the fetal brains from the WT and KO mice. Gene expression network analysis was performed as described in our earlier work [17,29].

### 2.5. Cell Type Deconvolution of Bulk RNA-Seq Data

The normalized gene expression, in TPM (transcripts per million reads), from the bulk RNA-seq data of the placentae and the fetal brains of the WT and KO mice were subjected to cell type deconvolution analysis. For each reference cell type of the placenta and the fetal brain, the expression level of the marker genes in TPM was generated from the snRNA-seq data using the *PseudobulkExpression* function of *Seurat*. The bulk RNA-seq data was deconvoluted to the reference cell types based on the TPM values of the markers genes by employing the *dtangle* method [33] implemented in the *R* package *granulator.*

### 2.6. Computational and Statistical Analysis

All computational analyses were performed using the University of Missouri high performance computing cluster *Lewis* via batch job submission through *Slurm*. All the statistical analyses and plotting were performed in *R*.

## 3. Results

### 3.1. Lack of Cav1 Impacts the Gene Expression of Placental Cells

**Figure 1:**
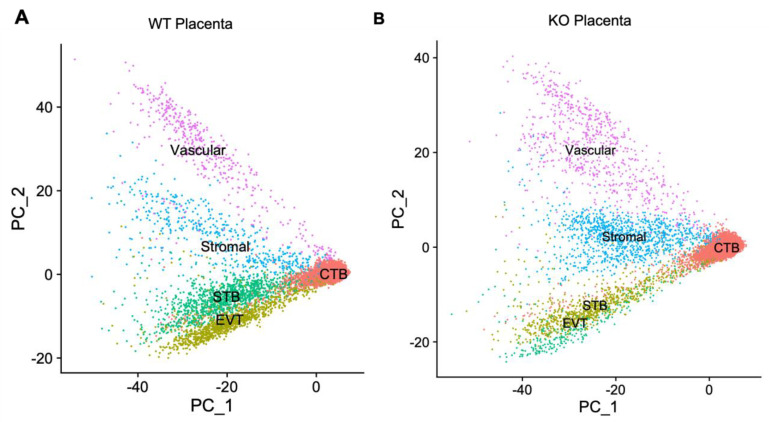
Cluster analysis of placenta snRNA-seq data shows the expression patterns of cytotrophoblast (CTB), syncytiotrophoblast (STB), extravillious trophoblast (EVT), stromal and vascular cells in WT (**A**) and *Cav1*-null (**B**) mice. The axes show the principal components of gene expression variation between cell types. The cell clusters are shown in different colors. Each dot represents a single cell.

A total of 22,138 and 23,099 nuclei were sequenced from the placentae of the WT and KO mice, respectively. Data analysis by *Seurat* [26] identified cytotrophoblast (CTB), syncytiotrophoblast (STB), extravillious trophoblast (EVT), stromal and vascular cells, with CTB being the most abundant cell type in both the WT and KO placentae (Figure 1). A nearly equal number of STB cells was observed in the WT and KO placentae, but the number of EVT cells in the KO mice was nearly 3-fold less compared to that of WT mice. In contrast, the stromal and vascular cells were, on average, 2-folds more abundant in the KO compared to the WT mice (Supplementary Figure S1).

### 3.2. Comparison of the Marker Genes of Placental Cells between WT and KO Mice

**Figure 2:**
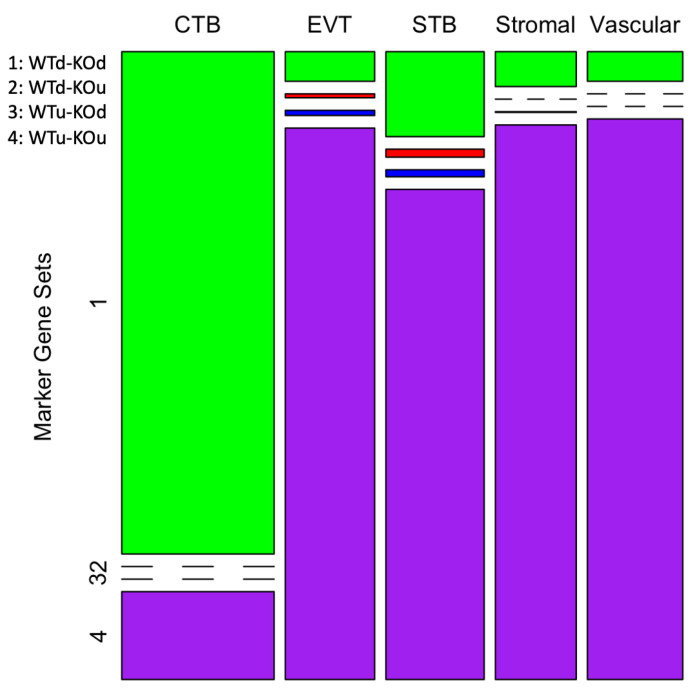
A mosaic-plot showing four patterns of gene expression in the placental cells. The columns represent the cell types and the rows represent the marker gene sets (1 through 4). The legends on the top left show the four groups of genes (u: upregulated, d: downregulated, WT: wild type, KO: knockout). The number of genes in each group is shown by the size of the color-coded boxes (or a line if the data point is zero).

Differential gene expression analysis by *Seurat* identified marker genes that were significantly differentially expressed (DE) in specific cell type(s) of the placenta between the WT and KO mice. Based on the direction of the differential expression (upregulation or downregulation) of the genes, four groups of DE genes were identified (Supplementary Table S1). The genes labeled as ‘WTd-KOd’ (Figure 2) were downregulated (d) in the CTB cells in both the WT and KO mice. A total of 3242 genes were identified in this group. On the other hand, the genes labeled as WTu-KOu were upregulated (u) in the STB, EVT, stromal and vascular cells (Figure 2). A total of 7191 genes were identified in this group (WTu-KOu). The chi-square test showed a statistically significant bias (chi-square 6062.94, *p* < 0.0001) in the number of genes belonging to these groups. The cytotrophoblast represents the stem cells of EVT and STB lineages [34]. It is known that genes with low expression in stem cells relative differentiated cells play important roles in cell differentiation [35]. In addition, we also identified genes, *albeit* fewer in number, that were either upregulated in the WT but downregulated in the KO mice (labeled as WTu-KOd) or vice versa (as WTd-KOu). The WTu-KOd (*n* = 42) and WTd-KOu (*n* = 39) genes were mostly observed in either the STB or EVT cells (Figure 2). These genes are also listed in Supplementary Table S1. No significant bias (chi-square 0.39, *p* = 0.53) was observed in the number of these genes differentially expressed between STB and EVT cells.

### 3.3. Genes Differentially Expressed in the STB and EVT Cells Due to Cav1 Ablation

**Figure 3:**
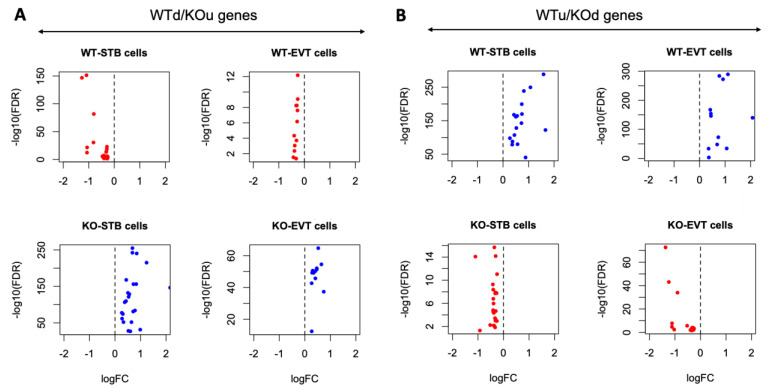
Contrasting patterns of gene expression in the EVT and STB cells of KO compared to the WT mice. (**A**) Gene expression pattern of WTd/KOu genes in the STB and EVT cells of WT and KO mice. (**B**) Gene expression pattern of WTu/KOd genes in the STB and EVT cells of WT and KO mice. In both (**A**,**B**), the log fold changes (x-axis) and log false discovery rate (y-axis) are plotted. The top panel represents WT mice and the bottom panel represents KO mice. Each dot represents a gene. A red dot means the gene is downregulated while a blue dot means the gene is upregulated in the cell types.

**Figure 4:**
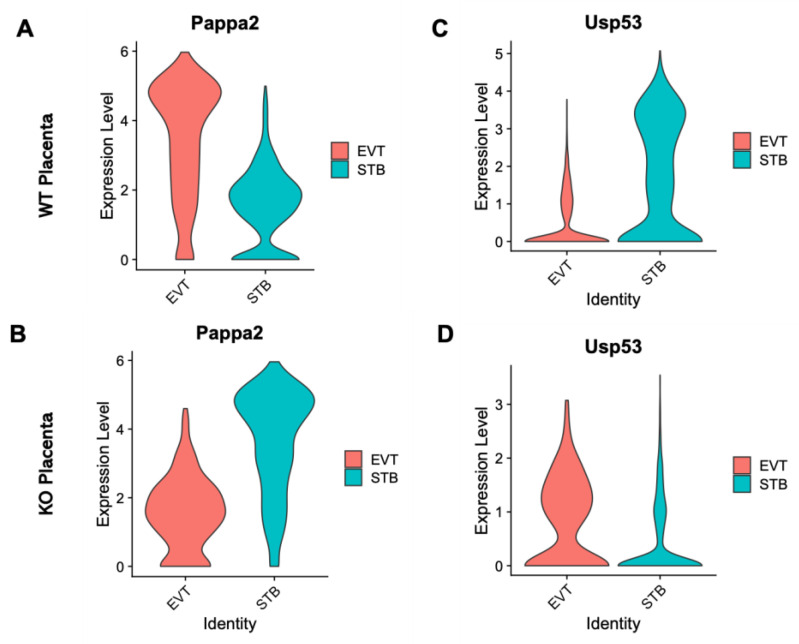
Violin plots showing the contrasting expression patterns of *Pappa2* and *Usp53* in the EVT and STB cells between WT and KO mice. (**A**) Expression pattern of *Pappa2* in the EVT (red) and STB (green) of WT mice. (**B**) Expression pattern of *Pappa2* in the EVT (red) and STB (green) of KO mice. (**C**) Expression pattern of *Usp53* in the EVT (red) and STB (green) of WT mice. (**D**) Expression pattern of *Usp53* in the EVT (red) and STB (green) of KO mice.

Specific genes (*n* = 81) were differentially expressed in the EVT cells relative to the STB cells due to the ablation of *Cav1* (Supplementary Table S1). The plots in Figure 3A show the upregulation of genes in the STB and EVT cells, and the plots in Figure 3B show an opposite pattern of gene expression due to the loss of *Cav1*. Of all the DE genes between the STB and EVT cells, pregnancy-associated plasma protein A2 (*Pappa2*), a gene known to modulate placental functions [36], and ubiquitin specific peptidase 53 (*Usp53*), another gene which is also linked to placental functions [37], showed the most fold changes but in opposite directions between the two cell types. *Pappa2* was upregulated in the STB cells compared to the ETV cells in the *Cav1* KO mice (Figure 4). But in the WT mice, the expression of *Pappa2* was lower in the STB cells compared to the EVT cells. On the other hand, *Usp53* showed higher expression in the EVT cells compared to the STB cells in the KO mice. But in the WT mice, *Usp53* showed an opposite expression pattern (Figure 4). We focused on STB and EVT cells in these analyses because these cells in the placenta play critical roles in the maternal–fetal communication [34]. STBs produce hormones that are necessary to sustain pregnancy and, at the same time, regulate the fetoplacental permeability that allows transfer of nutrient and gas to the fetus [34]. EVTs play a role in remodeling maternal spiral arterioles which allows blood supply to the fetus [38].

### 3.4. Deregulation of Cellular Crosstalk in the Placenta Due to the Absence of Cav1

**Figure 5:**
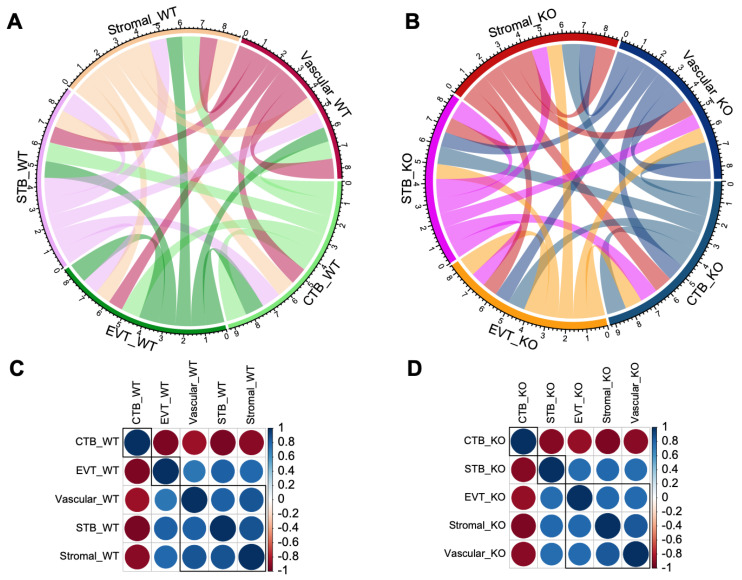
Patterns of transcriptional crosstalk among placental cell types in the WT and KO mice. The top panel (**A**,**B**) shows the Circos plots with colored tangles connecting between cell types. The connection pattern is based on the level of correlation in gene expression changes calculated in a pair-wise manner among the cell types. The plot in (**A**) represents the WT mice, and that in (**B**) represents the KO mice. The lower panel (**C**,**D**) shows correlation plots for gene expression changes among the cell types. The plot in (**C**) represents WT mice, and that in (**D**) represents the KO mice. Blue and red colored circles indicate a positive or negative correlation, respectively, in expression changes between cell types.

The patterns of transcriptional crosstalk among cell types were predicted by hierarchical clustering of gene expression variation between the cell types in the WT and KO mice (Figure 5A,B). In the WT mice, the stromal and vascular cells expressed genes in a more similar manner with the STB rather than the EVT cells (Figure 5C). But, in the *Cav1* KO mice, the stromal and vascular cells expressed genes in a more similar manner with the EVT rather than the STB cells (Figure 5D). Specific paralogs (duplicated genes) of the pregnancy-specific glycoprotein (*Psg*) gene family, such as *Psg16, Psg25, Psg26* and *Psg27*, showed relatively lower expression in the STB, stromal and vascular cells compared to the EVT cells in response to loss of *Cav1*. This is shown in the kernel density of gene expression pattern of the cells in the violin plots in Supplementary Figure S2. These results, consistent with our earlier finding about the expression of paralogous genes in the mouse placenta [6], suggested a potential role of gene duplications in the transcriptional crosstalk of placental cells in response to the loss of *Cav1*.

### 3.5. Impact of Cav1 Ablation on the Regulation of the Brain–Placental Axis

**Figure 6:**
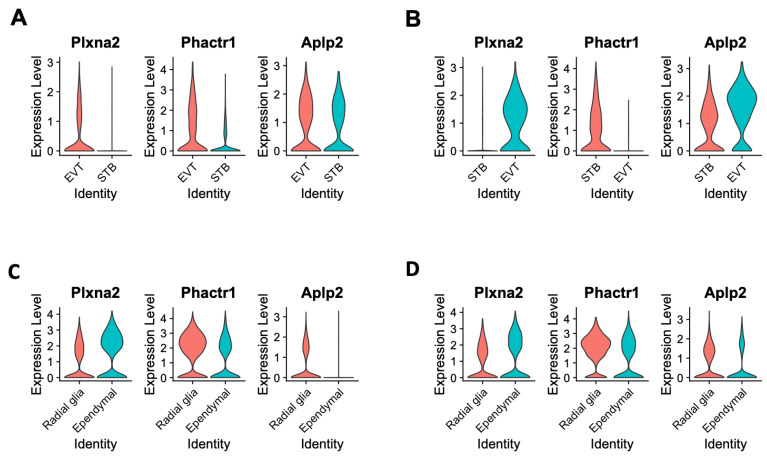
Violin plots showing the expression of plexin A2 (*Plxna2*), phosphatase and actin regulator 1 (*Phactr1*) and amyloid precursor-like protein 2 (*Aplp2*) genes in specific cell types of the placenta and the fetal brain. (**A**) Gene expression pattern in the EVT and STB cells of the placenta of WT mice. (**B**) Gene expression pattern in the EVT and STB cells of the placenta of KO mice. (**C**) Gene expression pattern in the radial glia and ependymal cells of the fetal brain of WT mice. (**D**) Gene expression pattern in the radial glia and ependymal cells of the fetal brain of KO mice. In each plot, the cell types are shown in the x-axis and the expression level is shown in the y-axis.

Gene Ontology (GO) enrichment analysis [39] showed that genes differentially expressed between STB and EVT cells due to the loss of *Cav1* were significantly enriched with functions related to neurogenesis, neuron differentiation and neuron projection development, among others. The fold changes of enrichment and the significance levels of these GO terms are provided in the Supplementary Table S2. The differentially expressed genes related to these significant GO terms are listed in the Supplementary Table S3. To further investigate regulation of these brain genes in the placental cells, the snRNA-seq data of the placenta generated in the present study were compared with the snRNA-seq data (accession number GSE214759) of the fetal brain generated from our recent study [17]. Of note, the fetal brain snRNA-seq data were generated from the same mice strains at the same gestation time as that of the placental snRNA-seq in the present study. The analysis identified specific genes such as amyloid precursor-like protein 2 (*Aplp2*), phosphatase and actin regulator 1 (*Phactr1*) and plexin A2 (*Plxna2*) that were differentially expressed in specific cell types of the placenta and the fetal brain due to the loss of *Cav1*. These genes were expressed differentially between EVT and STB cells in the placenta, and between radial glia and ependymal cells in the fetal brain (Figure 6). The *Aplp2* codes for the amyloid-beta precursor protein that produces amyloid-β toxin in humans with Alzheimer’s disease [40]. While the ablation of *Cav1* leads to molecular and cellular changes in mice that are the hallmarks of Alzheimer’s disease [18,41], the role of *Aplp2* in neurodegeneration in mice is not known. Rather, *Aplp2* is known to play a role in the survival and development of mice [42,43]. *Plxna2* is a key gene for axon guidance and brain development [44]. It is also active in the syncytiotrophoblast layer (SynTI) of the mouse placenta [45]. *Phactr1* functions in the cortical neurons during corticogenesis [46], and also as a regulator of endothelial cells where *Cav1* is abundantly expressed [47]. Regulation of *Aplp2* with *Phactr1* and *Plxna1* in specific cells of the placenta and the fetal brain indicates a role of these cells in the adaptive regulation of the brain–placental axis in the *Cav1*-null mice.

### 3.6. Bulk RNA-Seq and Deconvolution of Reference Cell Types in the Placenta and the Fetal Brain

**Figure 7:**
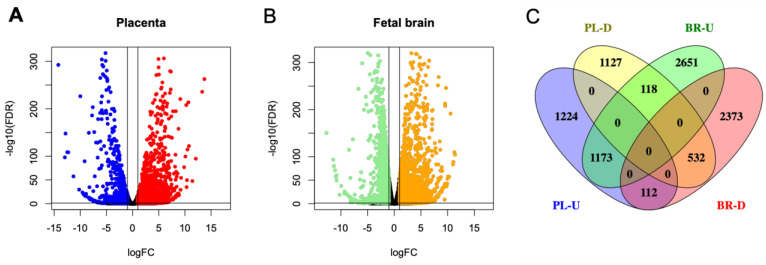
Differential expression (DE) patterns of the genes in the placenta and fetal brain based on bulk-RNA sequencing data. (**A**) Volcano plot showing upregulated (red) or downregulated (blue) genes in the placenta. (**B**) Volcano plot showing upregulated (orange) or downregulated (green) genes in the fetal brain. DE is calculated in KO relative to WT mice for placenta as well as fetal brain. Each dot represents a gene in both the plots. (**C**) Venn diagram showing the number of the specific or the shared DE genes in the placenta (PL) or fetal brain (BR) between the KO and WT mice.

**Figure 8:**
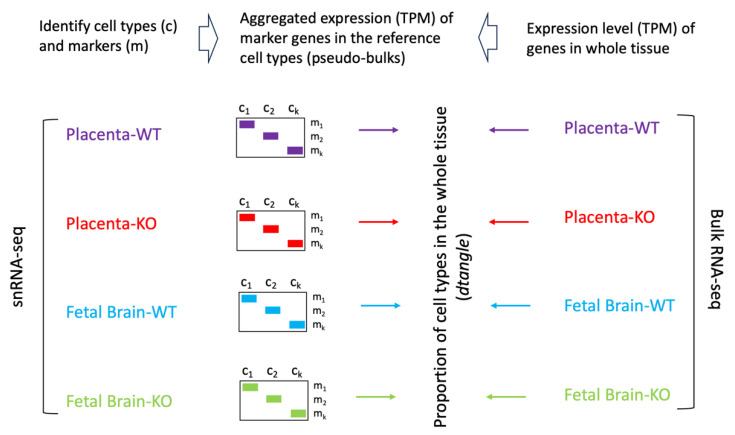
Workflow of comparing snRNA-seq and bulk RNA-seq data. After performing cluster analysis and annotating the cell types from the snRNA-seq data, aggregated read counts were generated for the marker genes for the individual cell types in the placenta and the fetal brain. These are referred to as the pseudo-bulk expression level of marker genes. The pseudo-bulk expression data were TPM (transcript per million) normalized. The read count data of the bulk RNA-seq data from the whole placenta and fetal brain samples were also TPM normalized for each gene. The bulk RNA-seq normalized data was then deconvoluted using the *dtangle* method (see main text) based on the pseudo-bulk expression data of the reference cell types to determine the proportion of cell types in the whole placenta and fetal brain samples.

**Figure 9:**
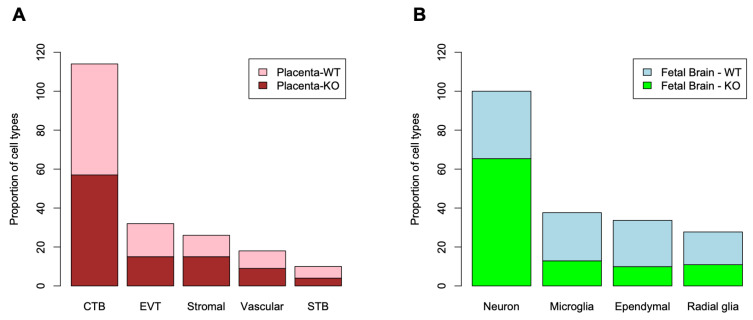
Deconvolution of bulk RNA-seq data for the proportion of reference cell types based on the snRNA-seq data. (**A**) The column graph shows the relative proportion of cell types (x-axis) in the placenta of WT and KO mice. Cell proportions are shown in y-axis. (**B**) The column graph shows the relative proportion of cell types (x-axis) in the fetal brain of the WT and KO mice. Cell proportions are shown in y-axis.

**Figure 10:**
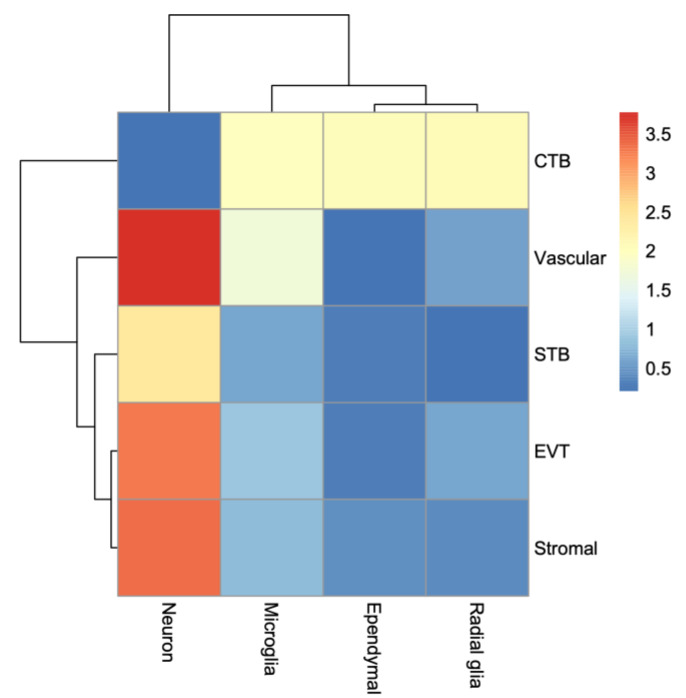
Heatmap showing patterns of gene expression changes between the placental and fetal brain cell types. The rows represent the cell types of the placenta, and the columns represent the cell types of the fetal brain. The variance of gene expression changes is color coded as shown in the scale bar on the right.

To assess the whole transcriptome of the placenta and the fetal brain, we performed bulk RNA-seq analysis (see Section 2). Differential expression (DE) analysis by *edgeR* [31] identified genes (*n* = 2509) significantly (False Discovery Rate or FDR < 0.05) upregulated in the placenta of KO compared to that of the WT mice (Figure 7A). But a relatively fewer number of genes (*n* = 1777) showed significant downregulation in the placenta due to *Cav1* ablation. In the fetal brain, 3952 genes were significantly (FDR < 0.05) upregulated whereas 3017 genes were significantly downregulated in the KO mice compared to the WT mice (Figure 7B). Each of these genes showed at least two-fold changes in expression, both in the placenta and in the fetal brain. The number of genes that were significantly altered, both in the placenta and the fetal brain, are shown in Figure 7C. A 2 × 2 contingency test showed a significant bias (*p* < 0.001) in the number of genes whose expression changed in the same direction in both the placenta and the fetal brain compared to the genes whose expression changed in an opposite direction. As *Cav1* is a regulator of cell proliferation and senescence [48], we wanted to know if loss of *Cav1* influenced the relative proportion of the cell types in the placenta and/or fetal brain. We addressed this question by the deconvolution of cell types from the bulk RNA-seq data of the placenta and the fetal brain based on the gene expression of the reference cell types in the snRNA-seq data (Figure 8). As snRNA-seq analysis is profiled typically from ~10,000 single nuclei isolated from the tissue sample, the deconvolution of bulk RNA-seq data based on the snRNA-seq data is essential to estimate the cell type proportions in the whole tissue [33]. We used a method called *detangle* which is a reliable and robust approach to calculate cell proportions by the deconvolution of bulk RNA-seq data [33]. The deconvolution analysis [33] showed that the loss of *Cav1* significantly altered (2 × 2 contingency test, *p* < 0.05) the relative abundance of stromal to EVT cells in the placenta (Figure 9A), and glial to neuronal cells in the fetal brain (Figure 9B). We then compared gene expression changes in the cell types between the placenta and fetal brain. The variance of gene expression changes (fold changes) showed a distinct cluster pattern between the placental CTB cells and neurons of the fetal brain (Figure 10). Because glia depletion is major cause of synaptic abnormalities and brain disorders [49,50], this finding suggested a possible role of glial depletion in the fetal brain in the premature aging and neurodegeneration in *Cav1*-null mice [18].

## 4. Discussion

This study was initiated to understand the molecular and cellular regulation of the fetoplacental communication in *Cav1*-null mice. In mice, loss of *Cav1* causes hyperproliferation and vascular abnormality [20]. During early stages of pregnancy, *Cav1* is tightly regulated in the uterus [51] likely to redistribute the uterine epithelial cells [52]. *Cav1* is abundantly found in the lipid rafts, called caveolae, of the plasma membrane. Though *Cav1* is abundantly present in the endothelial cells which are found in all tissues, it is also present in many other cell types [53]. Though *Cav1* is a part of caveolae, the function of *Cav1* does not depend on the caveolae. The caveolae-independent function of *Cav1* is also known [54]. Moreover, caveolae are present in the stromal and vascular cells but not in the trophoblast cells of the murine placenta [55]. Similar to placental cells, there are specific types of brain cells that either contain or do not contain caveolae. In the mouse fetal brain, there are no caveolae in the neuronal cells [56], but they are present in the glia cells [57]. *Cav1* also functions in a caveolae-independent manner during neuronal maturation in the developing brain of mouse [56].

The ablation of *Cav1* leads to different cellular abnormalities in mice [19,20,58,59,60]. Our recent study showed that the loss of *Cav1* impacted gene regulation within and between the neuronal and glial cells in the mouse fetal brain [17]. The present study shows that lack of *Cav1* impacts gene expression of the placental cells. By comparing the brain and placenta snRNA-seq data, we show that the ablation of *Cav1* impacts a set of genes in specific cell types in the placenta and fetal brain. The extravillous and syncytiotrophoblast cells were significantly impacted due to the absence of *Cav1*, and these cells are known to play vital roles in the fetoplacental communication during pregnancy. While the EVT cells invade the decidua to remodel the uterine spiral arteries [38], the STB cells are localized on the surface of villous trees in direct contact with the maternal blood to facilitate nutrient transfer from the mother to the fetus [34].

In the developing central nervous system, the differentiation of brain cells is accomplished by complex temporal and spatial cellular differentiation processes. On day 15, the placenta is fully functional [23] while the fetal brain shows the rapid growth of the choroid plexus and hypothalamus [10]. Soon after day 15, the fetal brain rapidly populates the neuronal cells of the hindbrain and GABAergic inhibitory neurons of the cortex, along with prominent growth of the pituitary stalk and the spinal cord columns.

Neurons generated in the fetal stage remain functional in the brain throughout life [61], although new neurons are also produced in the adult brain [62]. On the other hand, glia cells, including microglia and radial glia cells, have a reduced lifespan (i.e., higher turnover rates). In the placenta, the EVT and STB cells are also short-lived compared to the cytotrophoblast cells [63,64]. By comparing the snRNA-seq data of both placental and fetal brain cells, we observed that the loss of *Cav1* impacted specific cell types both in the placenta and the fetal brain. The CTB cells expressed genes at a significantly lower level compared to the differentiated cells (EVT and STB cells). This result is consistent to the idea that low gene expression in stem cells allows these cells to rapidly modulate the lineage programs for differentiation, a process called as ‘lineage priming’ [35]. The low level of gene expression in the CTB cells was unaffected by the deletion of *Cav1* suggesting its essential role in placental adaptation to modulate STB and EVT cells. This is also consistent with the finding that ablation of *Cav1* significantly deregulated the STB and EVT cells.

Data from the present study further showed that loss of *Cav1* altered relative proportion specific cell types in the placenta and the fetal brain. As shown in the violin plots in Figure 6, the kernel density of the plots varies between cell types that express *Plxna1*, *Phactr1* or *Aplp2*. This suggests that the genes are expressed in the placenta in a fewer number of STB cells but a greater number of EVT cells or vice versa. A similar pattern is also observed in the radial glia and ependymal cells of the fetal brain. Though in situ or immunohistochemistry can provide additional validation of the data (not generated in the current study), using these methods can be very challenging to discriminate gene expression at the single-cell level. However, recently, a single-cell in situ hybridization technique has been developed [65], and our future objective is to apply this approach to profile the spatial single-cell expression pattern of the brain and the placenta in future work. Moreover, *Cav1* is also a known regulator of cellular proliferation and senescence associated with aging-related diseases [66]. *Cav1*-null mice not only show neurodegeneration at an early age [18], but also show a drastically reduced lifespan [19]. We used bulk RNA-seq data to deconvolute [33] the proportion of reference cell types predicted from the snRNA-seq data. The EVT and stromal populations showed significant changes in cell proportions, but in an opposite direction, between the KO and WT mice. In the fetal brain, the proportion of neuronal and glial cells showed significant changes in relative proportion, also in an opposite direction, between the KO and WT mice.

Despite these important findings, our study has certain limitations. The present study did not determine if a decrease in EVT cell proportions due to the loss of *Cav1* impacted the placenta functions of supplying maternal nutrients necessary for optimal brain development. Therefore, the present study does not establish the causal effects of the placenta on the fetal brain. Our future plan is to address this limitation by developing a placenta-specific *Cav1* conditional knockout mouse model to investigate the direct role of placental *Cav1* on the development the offspring’s brain.

## 5. Conclusions

In conclusion, the results of this study show that specific cell types are associated with the transcriptional response of the placenta to the absence of *Cav1* in mice. Furthermore, the comparative analysis of gene expression changes in the placenta and the fetal brain suggests that *Cav1* plays an influential role in the regulation of the brain–placental axis in mice.

## Data Availability

The snRNA-seq data of placenta generated from this study are available in the Gene Expression Omnibus (GEO) database under accession number GSE248126. The bulk RNA-seq data generated from this study are available under accession number GSE248126.

## Supplementary Materials

The following supporting information can be downloaded at: https://www.mdpi.com/article/10.3390/cells13030215/s1, Figure S1: Differential proportion of EVT, stromal and vascular cells, but not STB cells, between WT (A) and KO (B) placenta; Figure S2: Violin plots showing expression changes of pregnancy-associated glycoprotein (Psg) genes among the placental cells. The cell types are shown in the x-axis and expression level is shown in the y-axis. The cell types are color coded; Table S1: List of marker genes differentially expressed in specific cell types of WT and KO mice; Table S2: List of significant GO terms; Table S3: List of genes differentially expressed in the EVT or the STB cells in the KO compared to the WT mice.
