# Supplementary material for: Single-Cell Transcriptional Response of the Placenta to the Ablation of Caveolin-1: Insights into the Adaptive Regulation of Brain–Placental Axis in Mice
Source: Cells. 2024 Jan 24;13(3):215. doi: 10.3390/cells13030215 (PMC10854826; doi:10.3390/cells13030215)
Supplement: Supplementary file 1 [file cells-13-00215-s001.zip › Supplementary Figures.pdf]

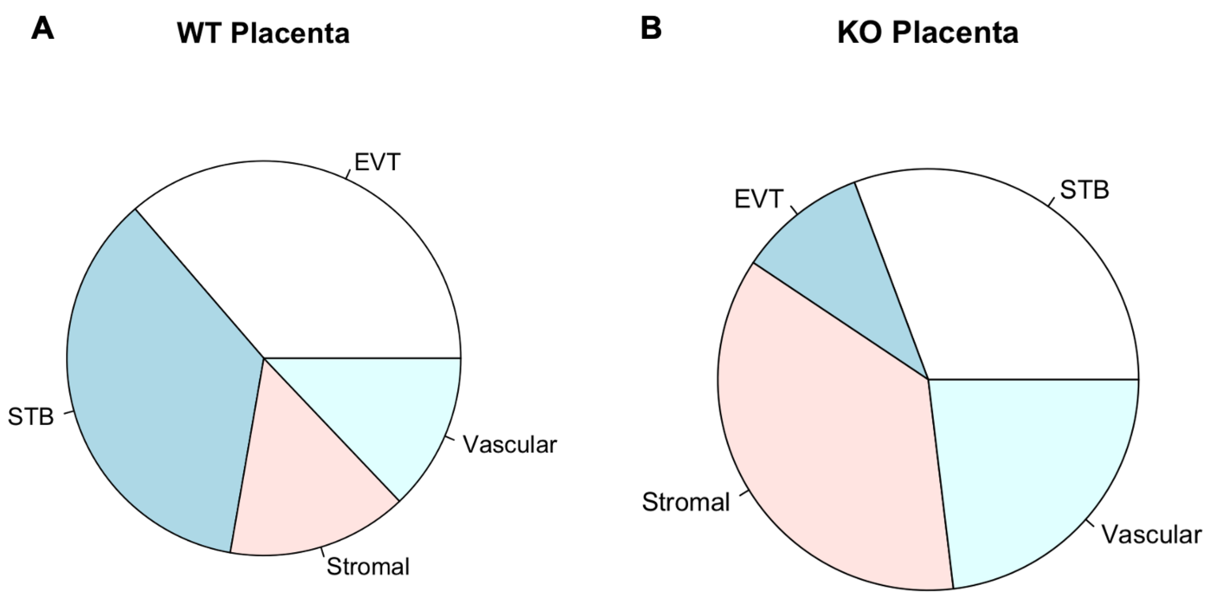

Supplementary Figure S1. Differential proportion of EVT, stromal and vascular cells, but not STB cells, between WT (**A**) and KO (**B**) placenta .

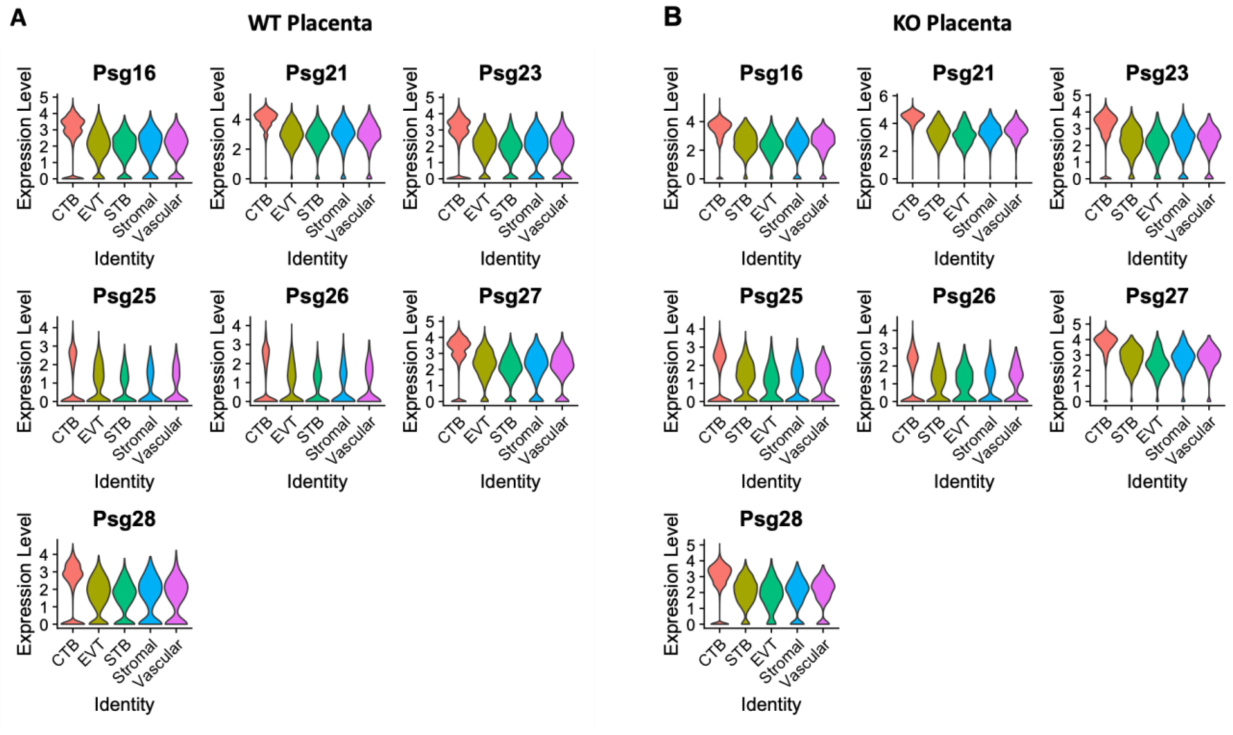

Supplementary Figure S2. Violin plots showing expression changes of pregnancy-associated glycoprotein (*Psg*) genes among the placental cells. The cell types are shown in the x-axis and expression level is shown in the y-axis. The cell types are color coded.
